# Primary hip and knee arthroplasty at district level is safe and may reduce the burden on tertiary care in a low-income setting

**DOI:** 10.1186/s12891-022-05936-z

**Published:** 2022-11-25

**Authors:** Kim Laubscher, Roopam Dey, Marc Nortje, Michael Held, Ntambue Kauta

**Affiliations:** 1 Orthopaedic Research Unit, Division of Orthopaedic Surgery, Department of Surgery, Groote Schuur Hospital, University of Cape Town, Cape Town, South Africa; 2 Investigation Performed at Groote Schuur Hospital and Mitchell’s Plain Hospital, Cape Town, South Africa; 3 Orthopaedic Biomechanics Lab, Division of Biomedical Engineering, Department of Human Biology, University of Cape Town, Cape Town, South Africa; 4 Mitchell’s Plain District Hospital, Cape Town, South Africa

**Keywords:** Arthroplasty, District hospital arthroplasty, Hip replacement, Knee replacement

## Abstract

**Background:**

Arthroplasty procedures in low-income countries are mostly performed at tertiary centers, with waiting lists exceeding 12 to 24 months. Recently, this is further exacerbated by the impact of the Covid Pandemic on elective surgeries. Providing arthroplasty services at other levels of healthcare aims to offset this burden, however there is a marked paucity of literature regarding surgical outcomes. This study aims to provide evidence on the safety of arthroplasty at district level.

**Methods:**

Retrospective review of consecutive hip and knee primary arthroplasty cases performed at a District Hospital (DH), and a Tertiary Academic Hospital (TH) in Cape Town, South Africa between 1^st^ January 2015 and 31^st^ December 2018. Patient demographics, hospital length of stay, surgery related readmissions, reoperations, post-operative complications, and mortality rates were compared between cohorts.

**Results:**

Seven hundred and ninety-five primary arthroplasty surgeries were performed at TH level and 228 at DH level. The average hospital stay was 5.2 ± 2.0 days at DH level and 7.6 ± 7.1 days for TH (*p* < 0.05). Readmissions within 3 months post-surgery of 1.75% (4 patients) for district and 4.40% (35) for tertiary level (*p* < 0.05). Reoperation rate of 1 in every 100 patients at the DH and 8.3 in every 100 patients at the TH (*p* < 0.05). Death rate was 0.4% vs 0.6% at district and tertiary hospitals respectively (*p* > 0.05). Periprosthetic joint infection (PJI) rate was 0.43% at DH and 2.26% at TH. The percentage of hip dislocation requiring revision was 0% at district and 0.37% at tertiary level. During the study period, 228 patients received their arthroplasty surgery at the DH; these patients would otherwise have remained on the TH waiting list.

**Conclusions:**

Hip and Knee Arthroplasty at District health care level is safe and; for the reason that the DH feeds into the TH; providing arthroplasty at district level may help ease the pressure on arthroplasty services at tertiary care facilities in a Southern African context. Adequately trained surgeons should be encouraged to perform these procedures in district hospitals provided there is appropriate patient selection and adherence to strict theatre operating procedures.

**Level of evidence:**

Level III Retrospective cohort study

## Introduction

Hip and knee replacement is amongst the most successful [1–4] and cost effective surgical interventions to increase quality-adjusted life expectancy (QALE) [3, 5, 6]. Despite this, even in developed nations a vast discrepancy exists between demand for surgery and service provision, resulting in lengthy waiting times [7–9].

This is perpetuated by depleted resources and an increasingly aging population [10], especially in low-middle income countries [11, 12], and furthermore exacerbated by the covid-19 pandemic [13, 14].

These economic barriers are also associated with a different comorbidity profile (i.e. HIV infection) compared to high income settings which could adversely affect outcomes [15].

A reasonable waiting time for orthopaedic elective surgery has previously been established at 10 weeks [16], although more than half of the patients experience further physical health deterioration during this waiting period [9]. Prolonged waiting times result in an increased cost burden as patients require additional analgesic medication and clinic visits with associated travel costs. This is often compounded by loss of work, especially in patients of low-income households [17]. In Southern Africa, arthroplasty is practiced both in state-funded tertiary centers and private hospitals. With limited access to private health care, state-funded hospitals are overburdened with waiting times of more than 12 months for arthroplasty procedures [7].

An expansion of arthroplasty services at a tertiary level may seem to be the obvious solution. But strategies to restrict arthroplasty to high-volume tertiary centres will notably affect older, poorer, less-educated, and rural patients, which will further widen existing economic disparities in arthroplasty surgery utilisation [18]. This has triggered arthroplasty surgery at more levels in the healthcare system including level 1 (district hospitals with limited specialist services) and level 2 (regional hospitals with at least two specialist services) [19].

When compared to tertiary care facilities, these first and second level hospitals may encounter increased challenges due to budget constraints and fewer qualified orthopaedic surgeons. Additionally, a higher proportion of presenting trauma-related surgical cases may affect DH resources for elective surgery. The resulting reduced volume in arthroplasty has been associated with inferior outcomes and a higher rate of complications [18, 20, 21]. Yet, to our knowledge no large study exists on the safety and complication rates for arthroplasty patients in district level facilities in a low-income setting [15, 22].

Our aim was to provide evidence on the safety of arthroplasty at district level and its potential to decrease waiting list burden in a low-income setting by describing the complication profile and surgical volume of arthroplasty surgery seen at the DH in relation to that seen at a tertiary care facility.

## Materials and methods

This is a retrospective comparative cohort study. Consecutive patients who underwent primary arthroplasty at an urban district hospital (DH) in Southern Africa from 2015 to 2018 were compared to those from a tertiary hospital (TH) in the same city. This DH is a direct referring facility to the TH. There is a 893 bed service in the TH (80 orthopaedics beds; and 4 dedicated high care beds for postoperative care); and 230 beds in the DH (of which only 18 are allocated orthopaedics beds). Both hospitals are state funded facilities. The DH services a population of approximately 1.2 million people with an average unemployment rate approximating 24.2%, providing primarily an orthopaedic trauma service with a limited hip and knee arthroplasty service [23]. The DH arthroplasty surgeries were evenly distributed among three specialist orthopaedic surgeons who shared 39 years of hip and knee arthroplasty experience (26, 11 and 2) All cases were discussed with the most senior surgeon and senior surgeon attendance was available whenever necessary. None of these three surgeons were performing surgeries at the TH. The TH has a dedicated arthroplasty unit with surgical team comprising two fellowship trained arthroplasty surgeons with an average of 10 years of arthroplasty experience (co-authors) each, and rotating trainee orthopaedic registrars. The registrars may perform certain surgeries but always under the direct supervision of the consultant. The primary author has worked at both DH and TH as trainee. All authors are part of the TH research unit.

Pre-operative American Society of Anaesthesiologists’ (ASA) scores were used as a simple guide to anaesthetic risk profile of the two cohorts [24]. Only patients with an ASA of I or II were selected for surgery at DH. The TH is able to cater for all ASA grades because of the availability of post-operative high care or intensive care unit.

The arthroplasty theatre complexes were equivalent in both hospitals, but laminar flow was only available in the TH operating rooms. Similar clinical pathways for arthroplasty surgery were used in both hospitals including antiseptic skin preparation, sterile surgical site draping and antibiotic prophylaxis as recommended by the NICE guidelines [25–28]. Patients received one dose of first-generation Cephalosporin antibiotic; or Clindamycin for those with penicillin allergy; prior to skin incision and 3 doses postoperatively. Surgical approaches included the medial parapatellar knee approach and either a posterior or anterolateral hip approach. All total knee implants were cemented, all total hip implants were uncemented. Wound closure was with sutures and local anaesthetic infiltration [29, 30]. Protocol for both DH and TH included graduated compression stockings, low molecular weight heparin (clexane) and physiotherapy commencing on day one post-surgery [30]. On discharge DH patients were started on low-dose aspirin for one month if no contraindications. TH patients did not receive any form of thromboprophylaxis post discharge. Patients were reviewed in the clinic at 2 weeks post discharge for wound check and removal of stitches, then at 6 weeks, 3 months, 6 months, 1 year and 2 years for clinical examination and follow up plain radiographs. Patients who completed an uneventful 2 year follow up were discharged from the service. Discharged patients had easy access back into the service should the need arise.

**Fig. 1:**
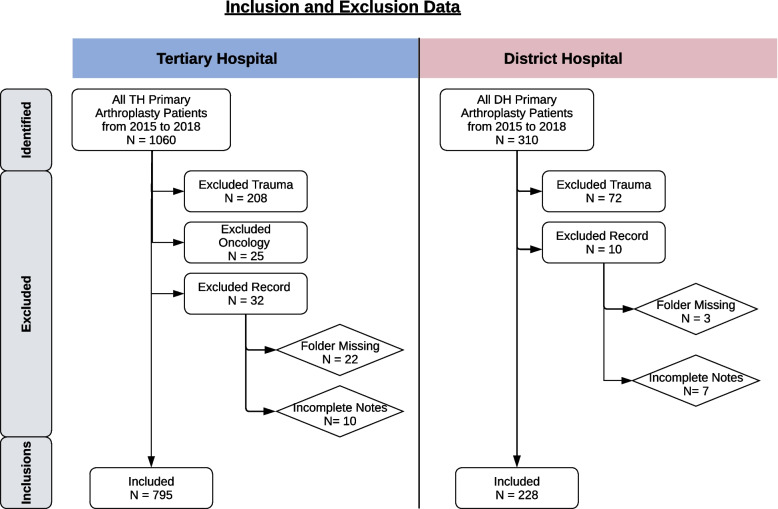
Inclusions and Exclusions Flow diagram

Ethical approval was obtained from the University of Cape Town Institutional Review Board South Africa (831/2019) prior to data collection, and the study was conducted in accordance with the Declaration of Helsinki.The arthroplasty service at the DH commenced in 2015. Therefore, all primary elective total knee and total hip replacement surgeries performed on skeletally mature patients in both the TH and DH, from 1 Jan 2015 to 31 Dec 2018 were included. Patients undergoing arthroplasty for trauma, or oncological conditions, as well as patients with missing or incomplete folders were excluded. (see Fig. 1).

Demographic data such as age and gender along with clinical information, such as HIV status, American Society of Anaesthesiologists (ASA) physical status classification [24], pathology, type of procedure, hospital length of stay, and post-operative complications were recorded.

Complications were categorised into orthopaedic and non-orthopaedic. Non-parametric continuous data was compared using Mann–Whitney U test. The dependence of categorical data on the hospital was analysed using a chi-squared test method. The statistical tests were performed in IBM SPSS v.27 (IBM, Armonk, NY, USA). Statistical significance was defined as *p*< 0.05. Strengthening the Reporting of Observational Studies in Epidemiology (STROBE) Statement guidelines were used to report the results [31].

## Results

During the study period, 795 tertiary level and 228 district level cases were included. Twenty-nine and a half percent of patients in the TH were male, compared to 27.4% at the DH (*p* = 0.53) (see Table 1).

**Table 1:** Patient demographics

**Characteristic**		**DH (*n* = 228)**	**TH (*n* = 795)**	
Average age		59.7 ± 10.9	60.1 ± 14.0	*p* = 0.17
Gender				
	Female	72.6%	70.5%	*p* = 0.53
	Male	27.4%	29.5%	
HIV Status	Positive	12.2%	3.4%	*p* = 0.000002
	Negative	23.5%	16.0%	
	Unknown	80.6%	64.3%	
American Society of Anaesthesiologists (ASA) physical status classification [24]				
	I	14.8%	12.3%	*p* = 0.0002
	II	74.3%	62.8%	
	III	10.9%	24.4%	
	IV	0.0%	0.5%	

At district level, a higher portion of ASA II patients (74.3% vs 64.1%) received surgery, whereas at the TH more ASA level III patients (23.4% vs 10.9%) were operated (*p* = 0.0002). A higher portion of HIV positive patients were operated in the district level hospital (12.2% vs 3.4%, *p* = 0.000002) although in most cases the HIV status was unknown.

**Fig. 2:**
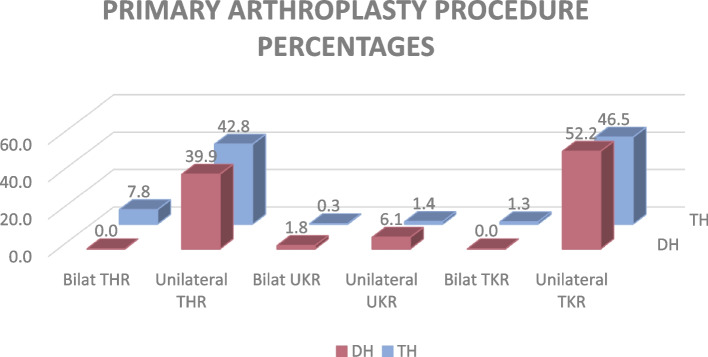
Type of Arthroplasty Procedure Performed

Primary unilateral Total Knee Replacement (TKR) was the most commonly performed surgery for both hospitals, accounting for 46.5% at tertiary level and 52.2% in district level (*p* = 0.001). Unilateral Total Hip Replacements (THR) accounted for 42.8% and bilateral THR 7.8% at TH level, while unilateral THR accounted for 39.9% (91) at DH level (see Fig. 2).

**Fig. 3:**
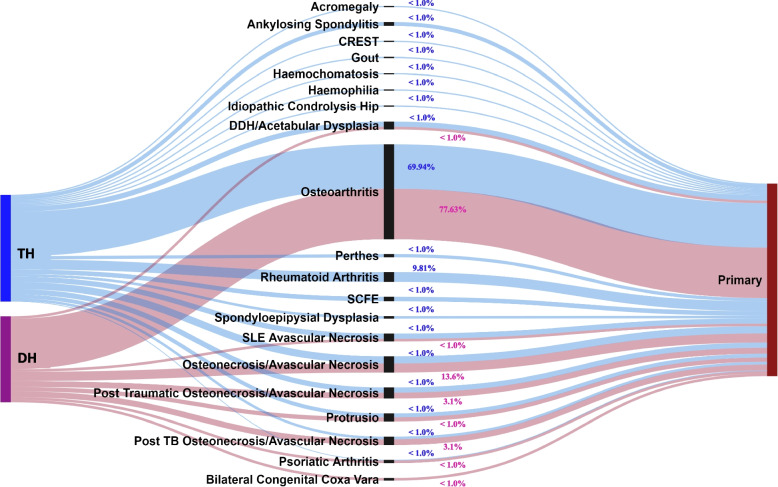
Aetiology

Primary osteoarthritis (OA) was the most common indication for arthroplasty surgery (77.6% in district level and 69.9% at tertiary level). This was followed by avascular necrosis (AVN) (13.6% and 10.7% in district and tertiary levels respectively) and post traumatic OA or AVN (3.1% vs 2.3%) *p* = 0.05. At tertiary level 9.81% of arthroplasty surgery was performed for rheumatoid arthritis (RA) while there were no cases of RA at district level. The remaining pathology/aetiology accounted for < 1% of surgeries. (See Fig. 3).

**Fig. 4:**
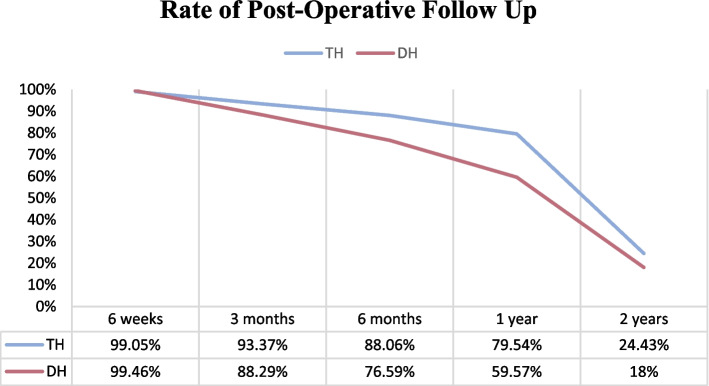
Rate of post-operative follow up

The follow up rate at post-operative week 6 was similar at both DH and TH level (99% of patients) but there were disparities at subsequent follow up visits (see Fig. 4).

Hospital stays for tertiary patients were longer than the district patients (7.6 ± 7.1 days vs 5.2 ± 2.0 days, *p* = 0.0001). The readmission rate at tertiary level was 4 in every 100 patients operated whereas at the district level the readmission rate was 1 in every 100 patients (*p* = 0.04). (see.

Tertiary Arthroplasty had 38.1% adverse events and district level had 32.6% (*p* = 0.01). Overall, physiological and mechanical complications predominated (10.8%) followed by non-orthopaedic related ward concerns (for example pressure sores, dizziness, confusion, glucose fluctuations, angina, constipation etc.) (4.8%) and wound related complications (3.6%).

Twenty-six patients (3.27%) in the tertiary hospital cohort received blood transfusion peri-operatively compared to 3 patients (1.31%) at the DH.

Reoperation rate of 1 in every 100 patients at district and 8.3 in every 100 patients at tertiary level (*p* = 0.04). Of the cases that required a second theatre episode at TH, 4.1% had revision arthroplasty and 4.2% for reasons other than revision; for instance; closed reduction of dislocated hip in theatre or manipulation under anaesthesia for stiff knee. (see Table 2).

**Table 2:** Comparison of orthopaedic outcomes

**Main Orthopaedic Outcome Measures**		**DH** **(*n* = 228)**	**TH** **(*n* = 795)**	
Number of Patients Requiring Revision		1.0% (3)	2.9% (23)	*p* = 0.04
Reoperation not for revision		0	4.2% (34)	*p* = 0.01
Dislocations		0	0.6% (5)	*p* = 0.14
	Multiple dislocations requiring revision	0	0.4% (3)	*p* = 0.29
PJI		0.87% (2)	3.5% (29)	*p* = 0.04
Superficial Infection		2.63% (6)	1.76% (14)	*p* = 0.05
Superficial wound problem other than infection		10.5% (24)	1.13% (9)	*p* = 0.42
Neurological Complications		2.6% (6)	1.8% (14)	*p* = 0.52
	Resolved	0.9% (2)	1.2% (10)	
	Persistent	0.9% (2)	0.4% (3)	
	Unknown	0.9% (2)	0.1% (1)	
Haemoglobin (g/dl)	Pre-operative Hb	13.0 ± 1.4 g/dl	12.8 ± 1.7 g/dl	*p* = 0.86
	Post-operative Hb	11.6 ± 1.7 g/dl	11.2 ± 1.8 g/dl	*p* = 0.02
	Average change in Hb	1.41	1.66	*p* = 0.01
Blood Transfusion		1.3% (3)	3.3% (26)	*p* = 0.12
Peri-Prosthetic Fracture		0.9% (2)	1.4% (11)	*p* = 0.67
Heterotrophic Ossification		0.9% (2)	1.8% (14)	*p* = 0.27

Table 3) Death rate was 0.4% vs 0.6% at district and tertiary hospitals respectively (*p* = 0.76).

**Table 3:** Comparison of non-orthopaedic outcomes

**Main Non-Orthopaedic Outcome Measures**		**DH (*n* = 228)**	**TH (*n* = 795)**	
Average Hospital Stay		5.2 ± 2.0 days	7.6 ± 7.1 days	*p* = 0.0001
	Knee	5.44	7.17	*p* < 0.001
	Hip	4.80	6.75	
Readmissions < 3mth postoperative		1.7% (4)	4.4% (35)	*p* = 0.04
Peri-operative Death		0.4% (1)	0.6% (6)	*p* = 0.76
Other infections e.g. UTI*, LRTI**		0.9% (2)	1.9% (15)	*p* = 0.27
Thromboembolic event		1.7% (4)	1.0% (8)	*p* = 0.52
	DVT	0.9% (2)	0.1% (1)	
	PE	0.4% (1)	0.6% (5)	
	CVA	0.4% (1)	0.2% (2)	
Anaesthetic Related Complications		0.4% (1)	2.5% (20)	*p* = 0.06

^*^UTI Urinary Tract Infection, **LRTI Lower Respiratory Infection

*DVT* Deep Vein Thrombosis, *PE* Pulmonary Embolis, *CVA* cerebrovascular accident

Revision surgery was done at both the DH and TH, with only one patient being referred from the DH to the TH for revision surgery. The mean number of days from primary surgery to time of first revision at TH was 283 days (IQR 22;392) and for DH 397 (IQR 28; 1099). The predominant reason for revision surgery was peri-prosthetic joint infections.

The predominant physical and mechanical complication recorded was leg length discrepancy. (see Table 4).

**Table 4:** Other physiological/ mechanical complications recorded

**Other Physiological/ Mechanical Complications**	**DH (*n* = 228)**	**TH (*n* = 795)**	
Leg Length Discrepancy (THR)	4.4% (10)	3.6% (29)	*p* = 0.19
Stiffness/ decreased ROM* (TKR)	2.6% (6)	3.3% (26)	
Fixed Flexion Deformity (THR)	1.0% (3)	1.4% (11)	
Patella Pathology (Mal-tracking/ Creps)	0.9% (2)	1.4% (11)	
Persistent pain > 3mths (TKR)	0.4% (1)	2.1% (17)	
Instability (TKR)	0.0% (0)	1.0% (8)	

^*^ROM Range of Motion

During the 4-year inaugural period of arthroplasty at the specific District Hospital studied, the arthroplasty service was able to process 22.3% (228 of the 1023 total cases done at these two facilities in the Western Cape) of the primary arthroplasty cases reviewed. This means that 228 patients received their arthroplasty at the DH; These patients would have been referred to the TH waiting list if DH offered no arthroplasty service.

## Discussion

Our findings show that hip and knee arthroplasty in our DH had a low complication rate, not only comparable to our TH, but also to other centres from high-income countries (HIC) [32–34]. [41],

A recent 10-week multicentred prospective observational study has also shown that arthroplasty at district hospitals and tertiary hospitals in South Africa had comparable peri-operative morbidity results as seen in our study [35].

The average length of stay at the TH (7.6 ± 7.1 days) was longer than at the DH (5.2 ± 2.0 days) with a *p* value of 0.0001. This is likely attributed to the fact that the TH catered for a higher proportion of patients with multiple co-morbidities and higher ASA grades, requiring multidisciplinary co-management and additional medical support.

In our study the mortality rate did not differ significantly between the two hospitals (DH 0.4% vs TH 0.6%; *p* 0.76) despite the differences in patients ASA grades in the 2 cohorts. The mortality rates are also comparable to international reports from HIC. The one-year mortality rates in the National Joint Registry for England and Wales (NJR) were 10.8 and 8.9 per 1,000 patient-years after hip and knee arthroplasty, respectively [32–34]. The cause of death at the district hospital was attributed to a thromboembolic event more than 2 weeks after surgery and to perioperative cardiorespiratory complications in patients with ASA grade III and IV at the TH. Although previous studies indicated the risk of mortality following surgery in patients across Africa is twice as high as the global average, this was not reflected in our study [36].

Our study showed an average blood loss of 1.41 g/dL and 1.66 g/dL for DH and TH respectively. This is consistent with the reported average decline in haemoglobin of 2.35 g/dL ± 1.14 and 2.29 g/dL ± 1.16 for THA and TKA [37]. In our TH, 26 (3.27%) patients received blood transfusion compared to 3 (1.31%) at the DH. This finding again may be a reflection of more complex cases (e.g., inflammatory arthropathies) being done at TH level with longer surgical time and possibly higher transfusion requirements.

The readmission rate of the TH was 4.40%, which was less than for the DH (1.75%), while still being within a range which is acceptable and reported in other centres [38]. Reasons for re-admission varied from infection, knee stiffness, wound-related problems, or cardiovascular complications. There were 3 TKR (1.04%) revision surgeries from district level. Reasons for revisions included aseptic loosening, early PJI and a polyethylene insert dislodgement.

At tertiary level 23 (2.9%) patients required revision surgery, staged procedures resulting in 35 revision cases done for the TH cohort. The most common indication for revision surgery was PJI. The higher percentage revision rate from the TH could be the result of more complex pathology as well as higher ASA grade of patients treated at the TH.

There were 11 (1.38%) peri-prosthetic fractures recorded in the tertiary group, 4 (0.50%) occurred intra-operatively, all during THR (2 femur and 2 acetabular fractures). The district cohort had 2 (0.87%) peri-prosthetic fractures, both intra-operative tibial fractures during TKR. Our cohort incidence of intraoperative periprosthetic fractures is comparable to that reported in the analysis of International Registry data (0.8%) by Pivec et al [39]. There were no dislocations recorded at district level, 5 (0.62%) at tertiary level. This falls within the acceptable range of 2—3% [40]. The rates of thromboembolic events recorded at TH and DH (1.01% and 1.75% respectively) were similar to those reported in the literature [38, 39]. (see Table 3) One thromboembolic event in each hospital resulted in death.

Leg length discrepancy after a THA is one of the major causes of patient dissatisfaction as demonstrated by Fujimaki et al [41]. Both hospitals showed analogous percentages of LLD; 3.65% (29) vs 4.38% (10) in tertiary and district level respectively. (see Table 4) However, a limitation of this study is that the change in leg length was not measured quantitatively but was rather a qualitative observation by the treating surgeons.

Overall tertiary and district level surgery had similar rates of physiological or mechanical concerns (11 vs 10%) (see Table 4), although many of these variables share the same concern as for LLD and were not measured comparisons.

The strength of this study is that it documents that an experienced surgeon using well accepted standards and techniques can perform TJA safely in a DH given careful patient selection (as per ASA classification and theatre protocols as discussed above). However, we must acknowledge its shortcomings, in that the study was conducted in a single district center with a relatively small sample size, and therefore results may not be generalisable or extrapolated to other district centers.

Only two state-funded facilities with a large discrepancy in the cohort sizes were assessed. Limited by the date of commencement of arthroplasty at the DH; the TH cohort had more than three times the number of patients. An important dispersion is generated, which makes it difficult to obtain statistically significant values. There was also no matching of cohorts to control for potential cofounders such as the discrepancy in comorbidity profile of patients. We were not able to include specific hip or knee scores or patient reported outcomes in our study outcome measures due to the retrospective nature of the study. Nevertheless, we can see that low-risk patients can safely have THR and TKR in the district setting provided the surgical methodology is up to the same standards as the TH. Another limitation is a relatively high loss to follow up; in low-income setting follow-up is often challenging and highlights the distances travelled by patients to access arthroplasty services [22].

## Conclusion

This study shows that primary elective hip and knee arthroplasty can be safely performed at a district level of care in a southern African setting. The morbidity and complication profile at the district level was similar to that seen at tertiary level of care and that reported in the literature. Arthroplasty practice at more levels of care will help decongest waiting lists at tertiary hospitals in our setting. Standard selection criteria and operating theatre protocols must be followed to optimise outcomes. Future studies should examine the capacity of the district facilities and how arthroplasty services could be optimised and upscaled to further aid southern African tertiary hospitals in reducing arthroplasty waiting times.

## Data Availability

The datasets generated and/or analysed during the current study are not publicly available due to patient privacy as well as data safeguard for the researchers but are available from the corresponding author on reasonable request.
